# Droitwich as a Health Resort

**Published:** 1905-01-07

**Authors:** 


### DROITWICH AS A HEALTH RESORT.

DROITWICH is in the county of Worcestershire, and is a little more than 120 miles from Paddington, whence it may be reached by quick train in about two and a half hours; the first-class return fare being 33s. 3d, and the second class 21s. The Brine springs have been known for a very long time, presumably as far back as the Roman occupation, for a colony was founded here and known as the Salinæ. When any place is very old there is always a wish to make it older still, and we are not greatly surprised to find that the Worcestershire Historian Habbington thinks that the "sprynges of salt" were there "from the tyme of Noe's Flud," and in Dome's Day Book the local men of position were allotted interest in the springs on condition that they supplied wood for fuel for the furnaces of the salt works.

The Brine river, which lies 200 feet below the level of the town, was in 1725 tapped by means of the Boring rod. This caused the springs to cease flowing, and now the Brine rises up the shafts to about 30 feet of the surface, and is then pumped into the baths and the salt works.

Not a few things have been vaunted as cures for cholera, and during the epidemic of 1831 baths of hot brine were tried. It is hard to say what amount of real success attended this practice; but, at any rate, it had the effect of bringing Droitwich into greater prominence, and soon afterwards the Royal Baths were founded. Later on more accommodation was called for, and the St. Andrew's Baths were opened. These are fitted with swimming, douche, needle, vapour, and ordinary baths. There is a staff of trained attendants for both ladies and men, and these attendants are competent to carry out massage, and also the Nauheim treatment for heart disease.

The treatment cannot well occupy more than a small portion of each day, and the invalids, or still more, those healthy people who accompany the invalids, often find the time hang heavily on their hands at many of these "cure" places. At Droitwich a good many pastimes and various recreations may be indulged in. There are hunting with the Worcestershire and other hounds, a nine-hole golf course near Dodder Hill, tennis, croquet, and various evening entertainments; and there is the pretty little park, with its daily summer concerts and band performances. Further afield many excursions may be made by train, carriage, or motor. An instructive day may be spent at the Worcester potteries; Gloucester, Hereford, Malvern, Tewkesbury, and Stratford-on-Avon are all accessible; and down the valley of the Wye Tintern Abbey and Chepstow may be visited. The town of Droitwich itself has some picturesque bits for the artist or photographer, and there are many short walks with nice views from the low-lying hills in the neighbourhood.

As to the diseases for which the Droitwich treatment is more especially suitable, we may mention gout as among those which is often unquestionably relieved. Of course in this disease something more is required than any form of medicinal treatment, and the patient who sighs for a "cure," but who will not restrain himself had better stay at home or keep this fact in view. In some forms of rheumatism, rheumatic arthritis, sciatica and lumbago, the brine baths and concomitant treatment very often bring relief and sometimes cure.

For some years the Nauheim or Schott treatment for heart disease has been carried out here; and from our own observations we should say that the Droitwich brine is quite equal to that of Nauheim in its curative properties; and certainly the gymnastic exercises can be just as well employed in Droitwich as anywhere else. And here, as at

Nauheim, some of the sufferers from heart disease have benefited enormously by the treatment.

Dr. Roden, of Droitwich, has written a very useful pamphlet on the Brine Baths, and refers to the diseases in which he believes the treatment is most efficacious. He also gives an analysis of the water which we see contains chloride of magnesium, sulphate of lime, sulphate of alumina, sulphate of soda, and, of course, a very large amount of common salt, making the waters of Droitwich "the strongest which are as yet known." The charges for private baths are from 1s. to 2s. 6d. at the St. Andrew's Baths, and we believe they are somewhat cheaper at the Royal, and at both a reduction is obtained by taking a series of tickets.

The hotels are the Worcestershire and the Raven, both quite close to the St. Andrew's Baths; the Royal, recently enlarged, is close to the Royal Baths, and the Park Hotel, about midway between the two bathing houses. There is also a hydropathic establishment and there are several boarding houses; for instance the Pembroke, Randolph, Clarendon, etc.

